# Effectiveness of a standardized scenario in teaching the management of pediatric diabetic ketoacidosis (DKA) to residents: a simulation cross-sectional study

**DOI:** 10.1186/s12909-024-05334-0

**Published:** 2024-03-27

**Authors:** Alice Monzani, Elena Corti, Antonio Scalogna, Silvia Savastio, Erica Pozzi, Pier Paolo Sainaghi, Francesco Della Corte, Ivana Rabbone

**Affiliations:** 1 Division of Paediatrics, Department of Health Sciences, University of Piemonte Orientale, via Solaroli 17, 28100 Novara, Italy; 2 SIMNOVA Interdepartmental Centre for Innovative Learning and Simulation in Medicine and Allied Health Professions, University of Piemonte Orientale, via Lanino 1, 28100 Novara, Italy; 3 Department of Translational Medicine, University of Piemonte Orientale, via Solaroli 17, 28100 Novara, Italy

**Keywords:** Diabetic ketoacidosis, Pediatrics, Simulation

## Abstract

**Background:**

Diabetic ketoacidosis (DKA) is a frequent manifestation at the onset of type 1 diabetes mellitus in children, possibly associated with a wide range of complications, often as a consequence of wrong or delayed treatment. Due to its complex and risky management, direct exposure to real situations alone is not sufficient to achieve adequate skills in pediatric DKA for residents. Simulation could be a valuable aid, allowing to practice a standardized scenario of a complex real-world situation. We aimed to test the effectiveness of a standardized scenario of pediatric DKA in teaching its recognition and treatment.

**Methods:**

We develop a standardized scenario able to guide step-by-step the learners through the flowchart of DKA management and considering alternative evolutions in the case of possible deviations from guidelines. It was a real-life simulation with the use of a high-fidelity pediatric simulator. It was played by 78 pediatrics 20 and emergency medicine residents. At the end of the simulation, a validated questionnaire was administered to collect feedback from participants regarding the impact of the simulation on learning. All materials to reproduce the DKA scenario are provided.

**Results:**

Overall, the scenario was rated as realistic (mean score 4.37 ± 0.68, from 1 to 5) and relevant to professional training (4.72 ± 0.47), useful in increasing confidence in interpreting laboratory tests (3.97 ± 0.65), group organization and communication strategies (3.49 ± 0.94), and managing the treatment of DKA (3.46 ± 0.92).

**Conclusions:**

The use of a standardized scenario of pediatric DKA may be a valid tool to reinforce theoretical knowledge in residents, both in pediatrics and in emergency medicine, and to directly and safely practice pediatric DKA management.

**Supplementary Information:**

The online version contains supplementary material available at 10.1186/s12909-024-05334-0.

## Background

Diabetic ketoacidosis (DKA) is the most common emergency related to acute hyperglycemia in patients with type 1 diabetes mellitus (T1DM), being the leading cause of morbidity and mortality in these patients. It is characterized by a severe metabolic derangement, due to the lack of insulin and increased circulating counter-regulatory hormones, which progressively leads to acidosis and dehydration, eventually evolving to coma and death if not promptly and adequately treated [1, 3].

The diagnosis of DKA is based on the presence of hyperglycemia (blood glucose > 11 mmol/L or 200 mg/dL), ketosis (serum β-hydroxybutyrate concentration > 3 mmol/L, or ketonuria ≥ 2+), and metabolic acidosis (pH < 7.30 or serum HCO3- < 18 mmol/L), accompanied by a varying degree of hypovolemia [2–4].

The frequency of DKA at T1DM onset varies approximately between 15% and 70% in Europe and North America. The risk of DKA in patients already diagnosed with T1DM ranges from 1 to 10% per patient per year [2–5].

Fluid and electrolyte administration and insulin therapy are the basic steps of DKA therapy. The goal is to correct acidosis, ketosis, and electrolyte imbalance, restore normal circulatory volume and blood sugar level, and avoid possible complications of DKA [2–4]. Indeed, DKA is associated with a wide range of complications, if not properly and timely managed. Among them, hypokalemia or other electrolyte changes, hypoglycemia, and more fearsome, cerebral edema, which in its severe form occurs in 0.3–0.9% of pediatric DKAs. Cerebral edema has a high mortality (21–24%) and permanent neurological morbidity (20–26%), being responsible for 70–80% of DKA deaths [2–4]. Management of young patients with DKA should occur in a hub center experienced in pediatric DKA or, if not possible, arrangements should be made to contact a physician experienced in DKA [3, 4, 6]. The first assessment of pediatric patients with DKA is frequently performed in the emergency department, where not only pediatricians but also emergency medicine physicians may be involved. The proper management of DKA is outlined by national and international recommendations [3, 4, 7]. Proper application of guidelines in emergency situations can be complicated and error-prone because they are highly time-dependent and due to the emotional burden and different management of pediatric DKA compared with adults [2, 4, 8]. Nonetheless, only strict adherence to DKA management guidelines can reduce deviations and errors, related to a worse clinical outcome [3, 6, 9].

For these reasons, simulation could be a valuable aid, allowing to practice a standardized scenario of a complex real-world situation, with the purpose of facilitating learning through immersion, reflection, feedback, and practice, without the risks that such a situation would entail in reality [8, 10, 11]. In this study, we tested the effectiveness of a standardized scenario of pediatric DKA that can be used to improve its recognition and treatment, according to current international guidelines [3].

## Methods

A standardized simulation scenario was developed with the collaboration of pediatric diabetologists, pediatric simulation experts, and medical simulation technicians. The scenario was constructed based on real cases, reworked according to teaching needs, learning, and educational objectives (Appendix A) (see Video, Additional file 2, which shows the setting of the scenario). All subjects provided written informed consent to participate to the simulation study and to the tape-recording of scenarios and their publication. The need for ethics approval was deemed unnecessary by the local Ethics Committee (Comitato Etico Territoriale Interaziendale AOU Maggiore della Carità di Novara) because the study has negligible risks involving only health care professionals for education purposes and not involving patients.

The simulations were performed by pediatrics and emergency medicine residents of University of Piemonte Orientale to evaluate its appropriateness and teaching effectiveness. All participants (both pediatrics and emergency medicine residents) attended a theoretical lesson about the principles of pediatric DKA management in the past 6 months. The simulation sessions occurred at the Interdepartmental Centre for Innovative Didactics and Simulation in Medicine and Health Professions of the University of Piemonte Orientale (SIMNOVA) from January to June 2022. The materials, environment, and personnel involved in the simulation are detailed in Appendix B. Before starting the simulation session, participants were divided into teams of 3 members. All teams attended the same introductory briefing in which they were shown the simulation environment, the high-fidelity simulator (SimJunior, Laerdal, Wappingers Falls, NY, USA), and the available equipment. Each team was given a few minutes to organize roles, then they were given the initial information regarding the patient and the simulation was started (the scenario is described in appendices C, D, and E).

Participants were expected to collect the patient’s history, perform a physical examination, and request appropriate laboratory tests to make a diagnosis of DKA and initiate the appropriate treatment. Laboratory results, chest X-ray, ECG were provided upon participants’ request during the scenario, according to the performed actions. The examination results are shown in Figs. 1–11.

**Fig. 1:**
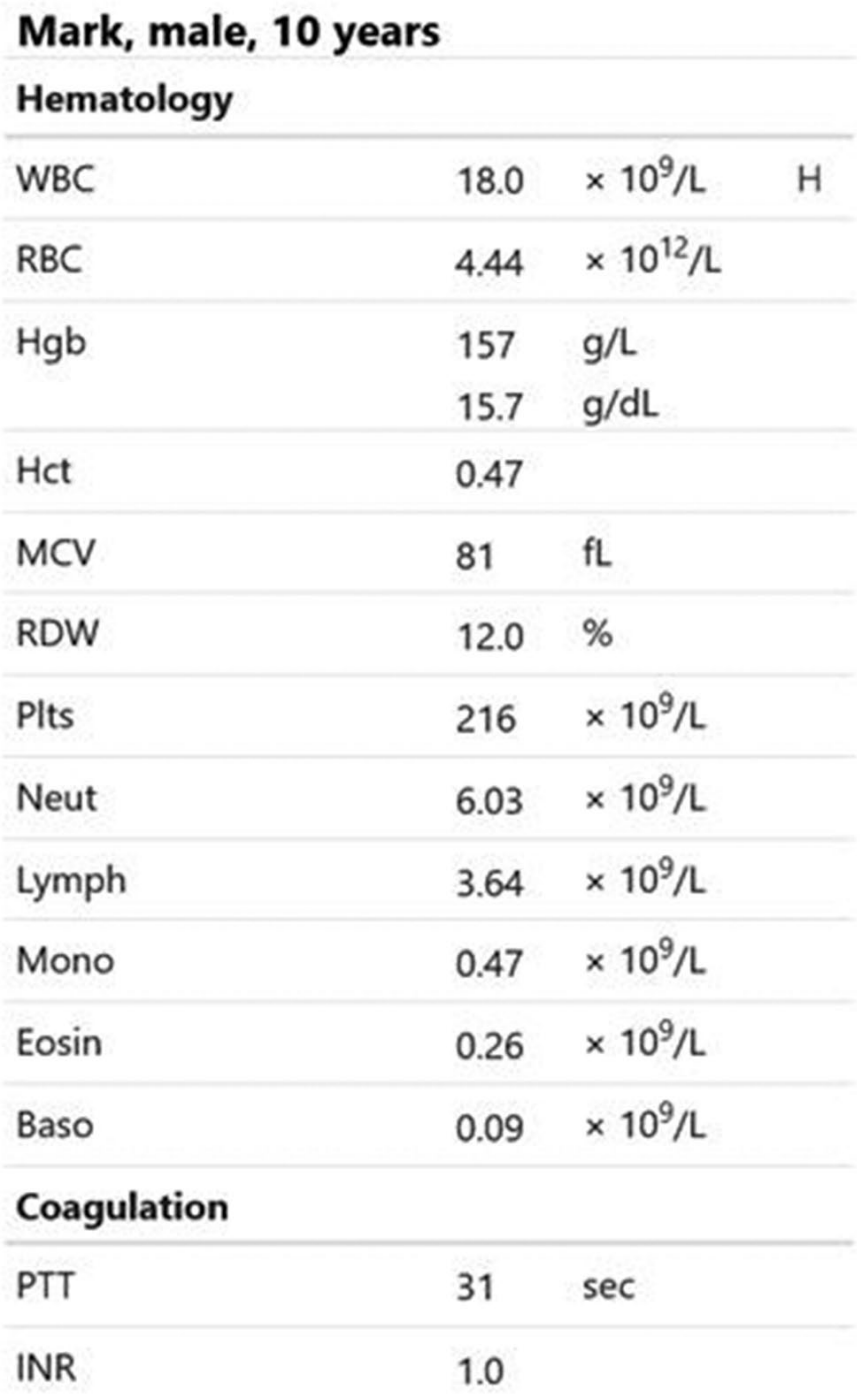
Blood count and coagulation at T0

**Fig. 2:**
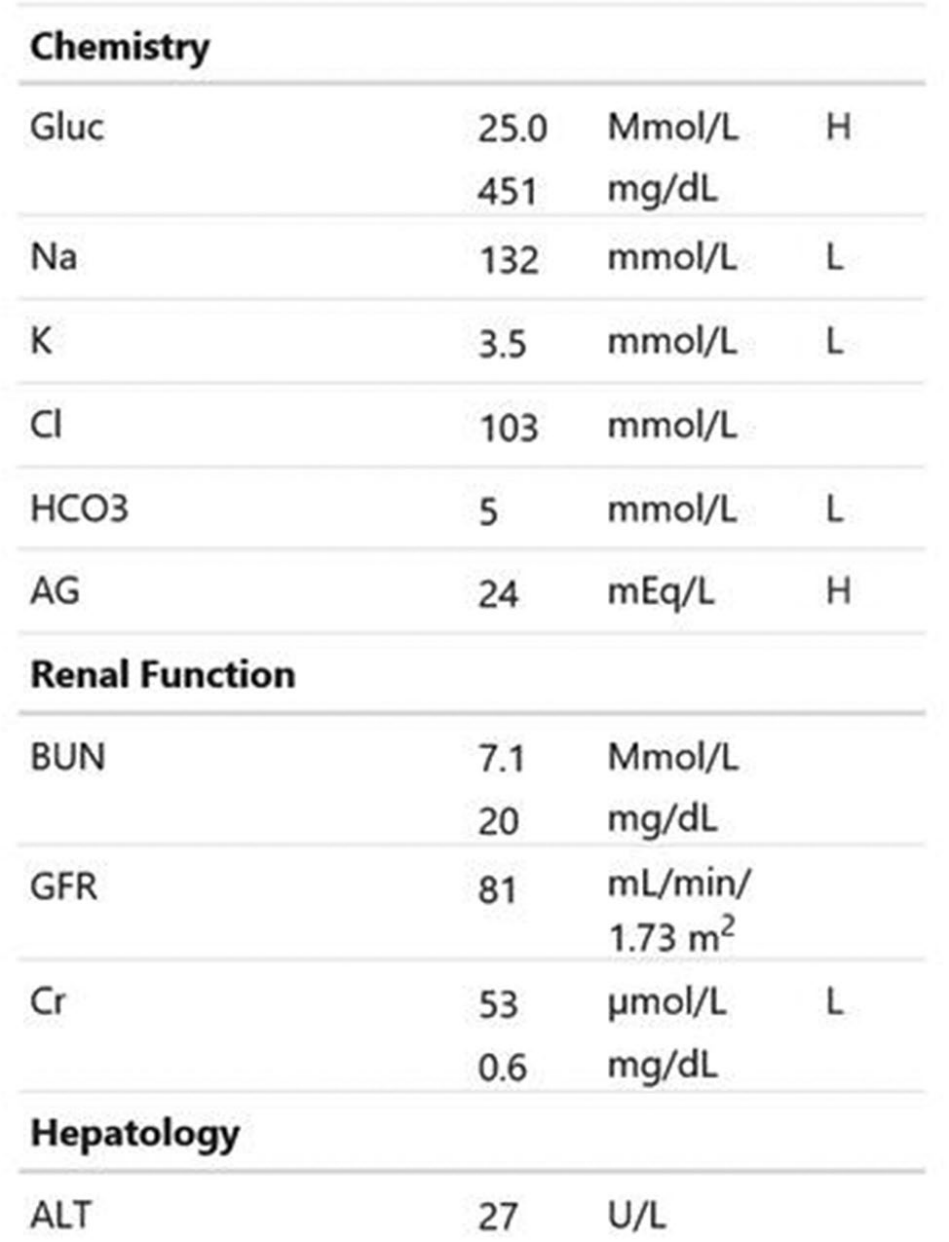
T0 biochemistry results

**Fig. 3:**
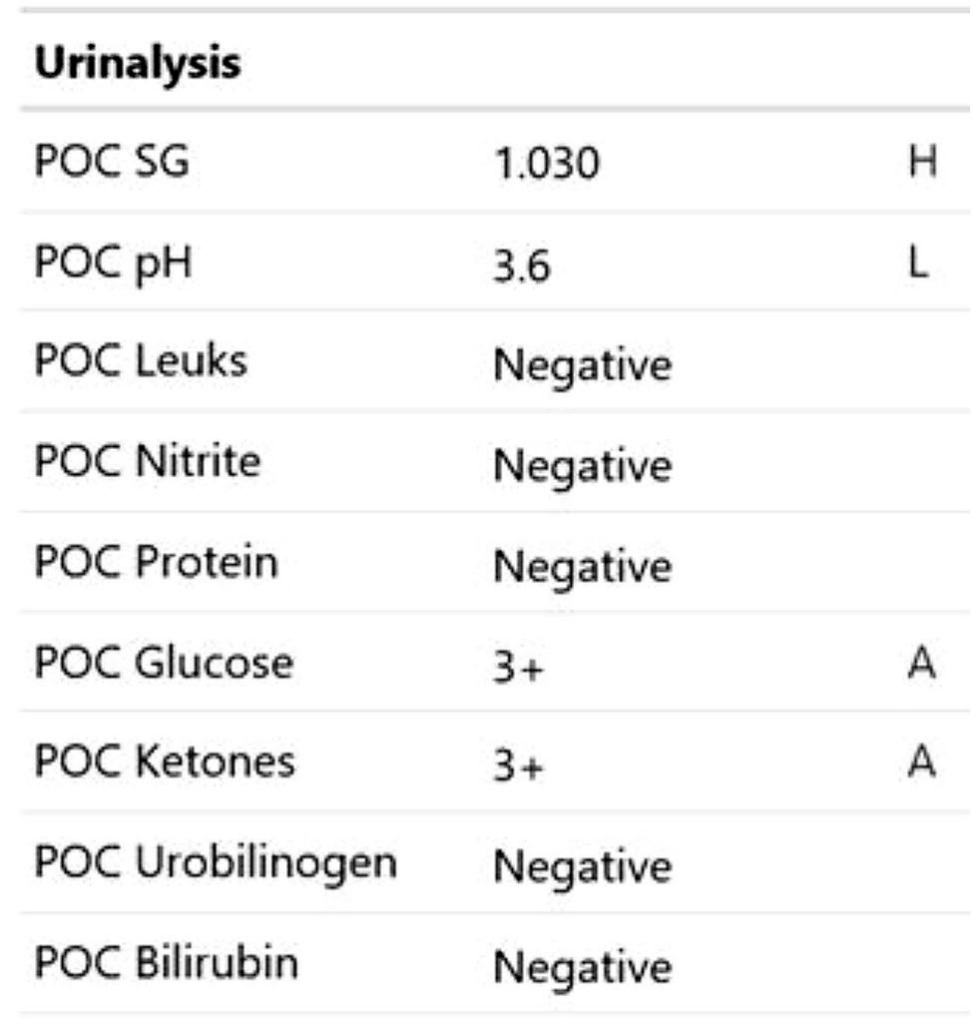
T0 Urine analysis

**Fig. 4:**
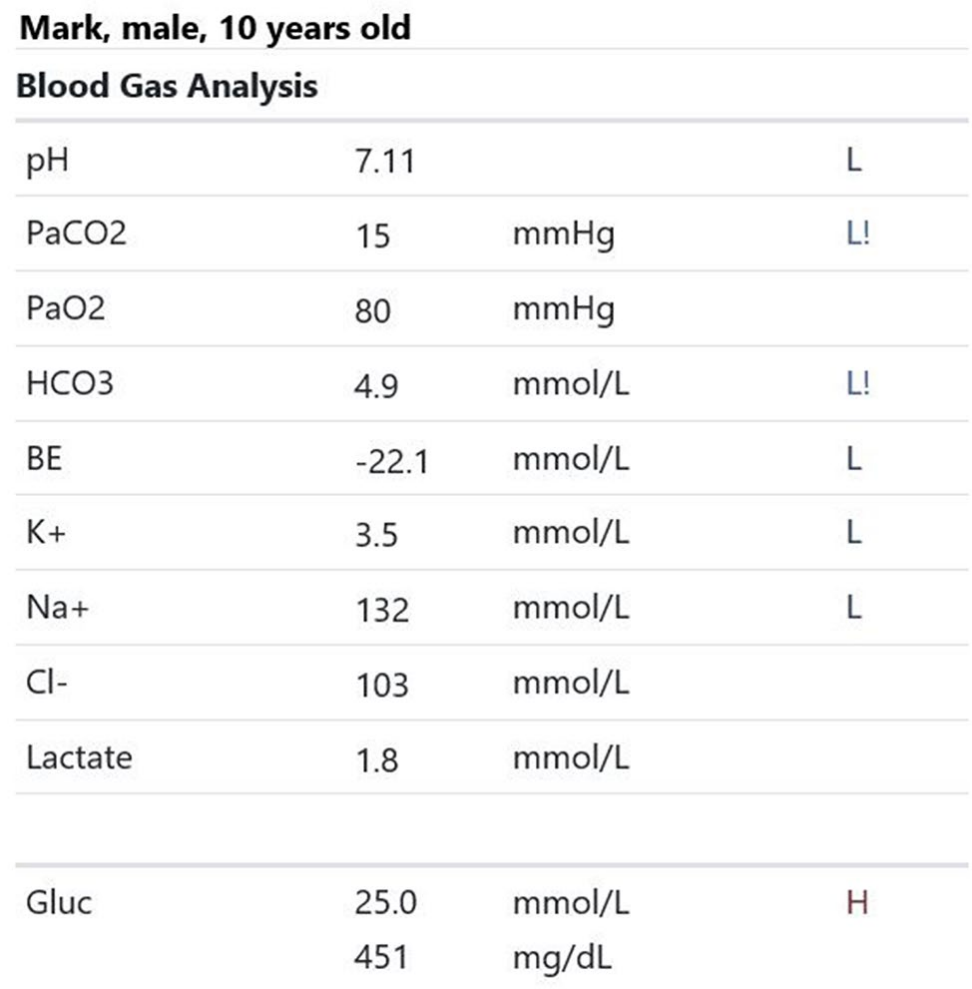
T0 blood gas analysis

**Fig. 5:**
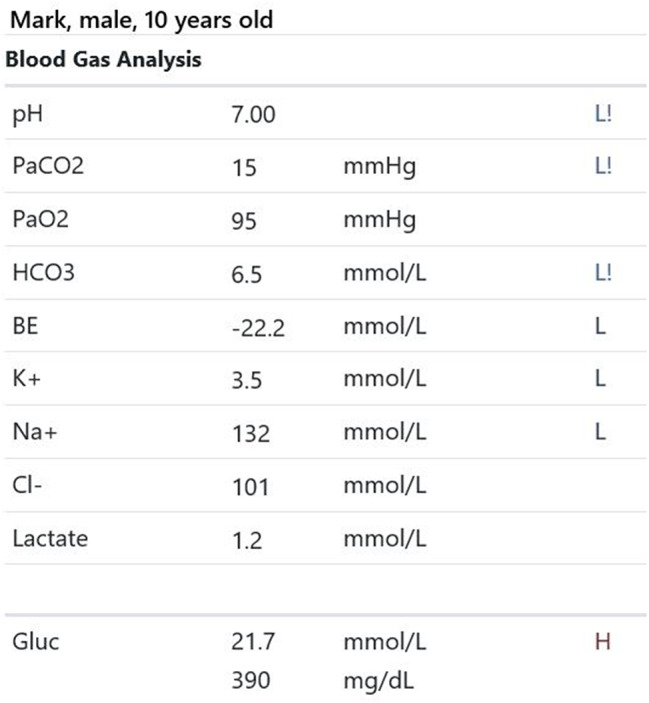
T2 blood gas analysis

**Fig. 6:**
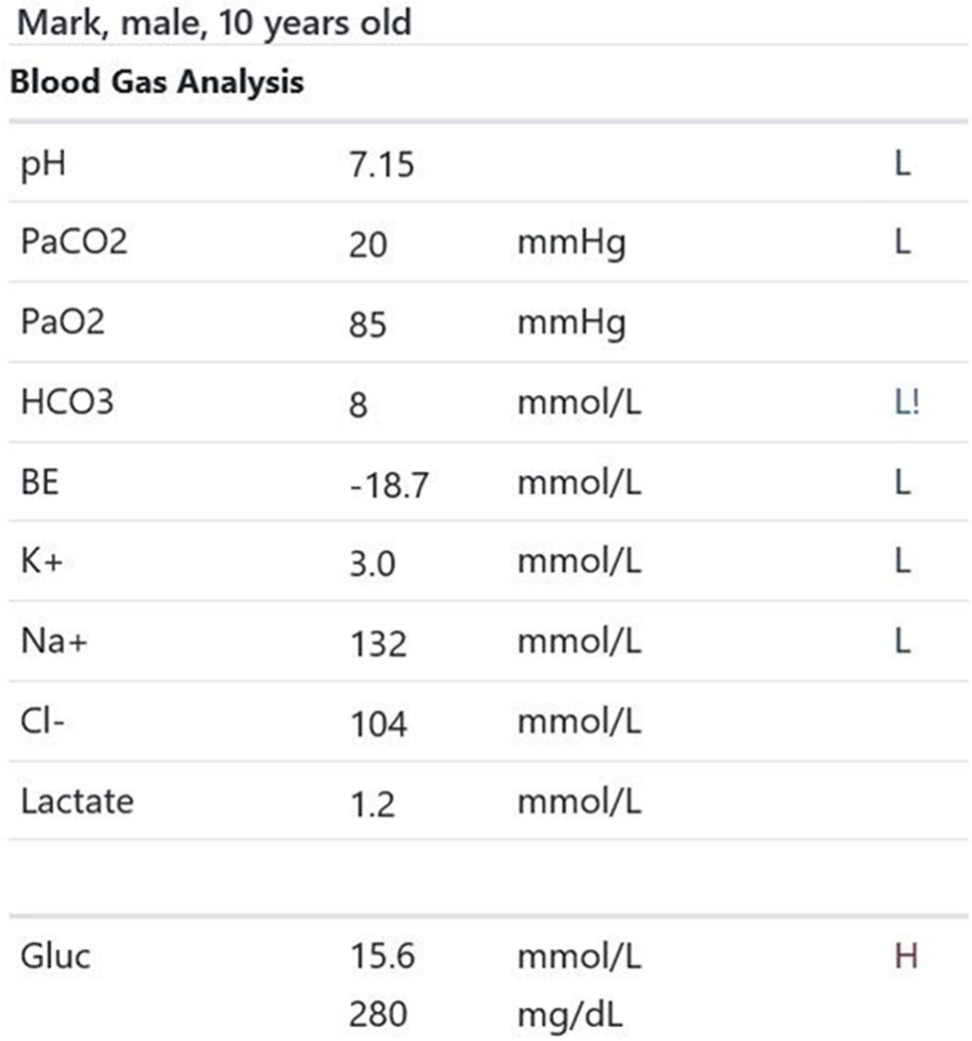
T4 blood gas analysis

**Fig. 7:**
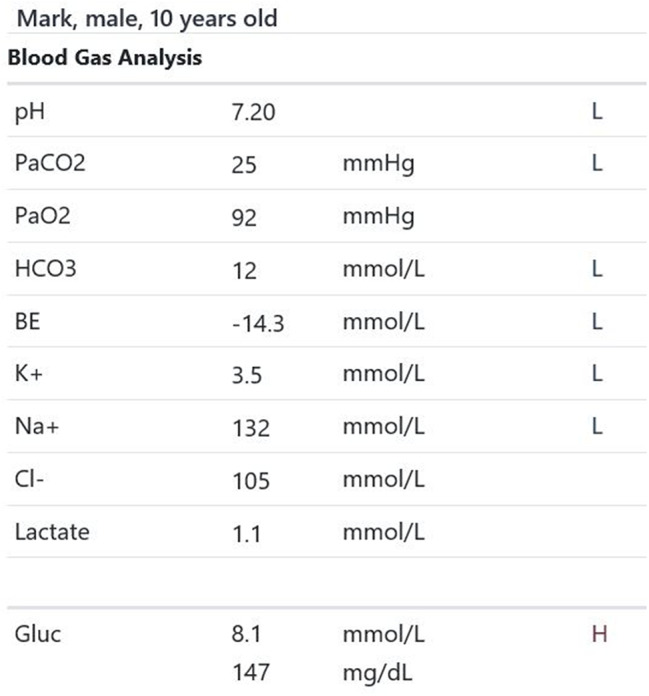
T6 blood gas analysis

**Fig. 8:**
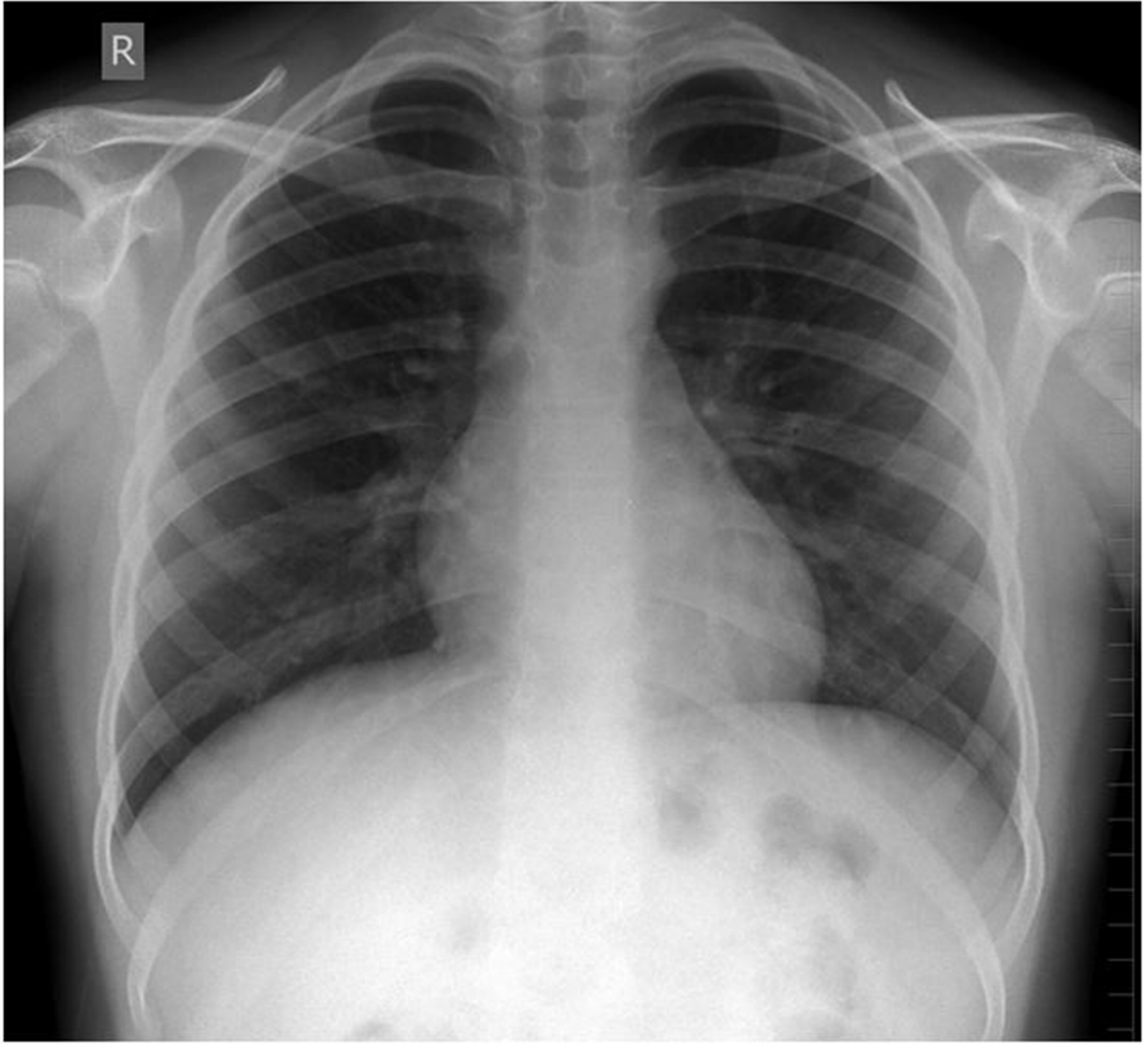
Chest X-ray

**Fig. 9:**
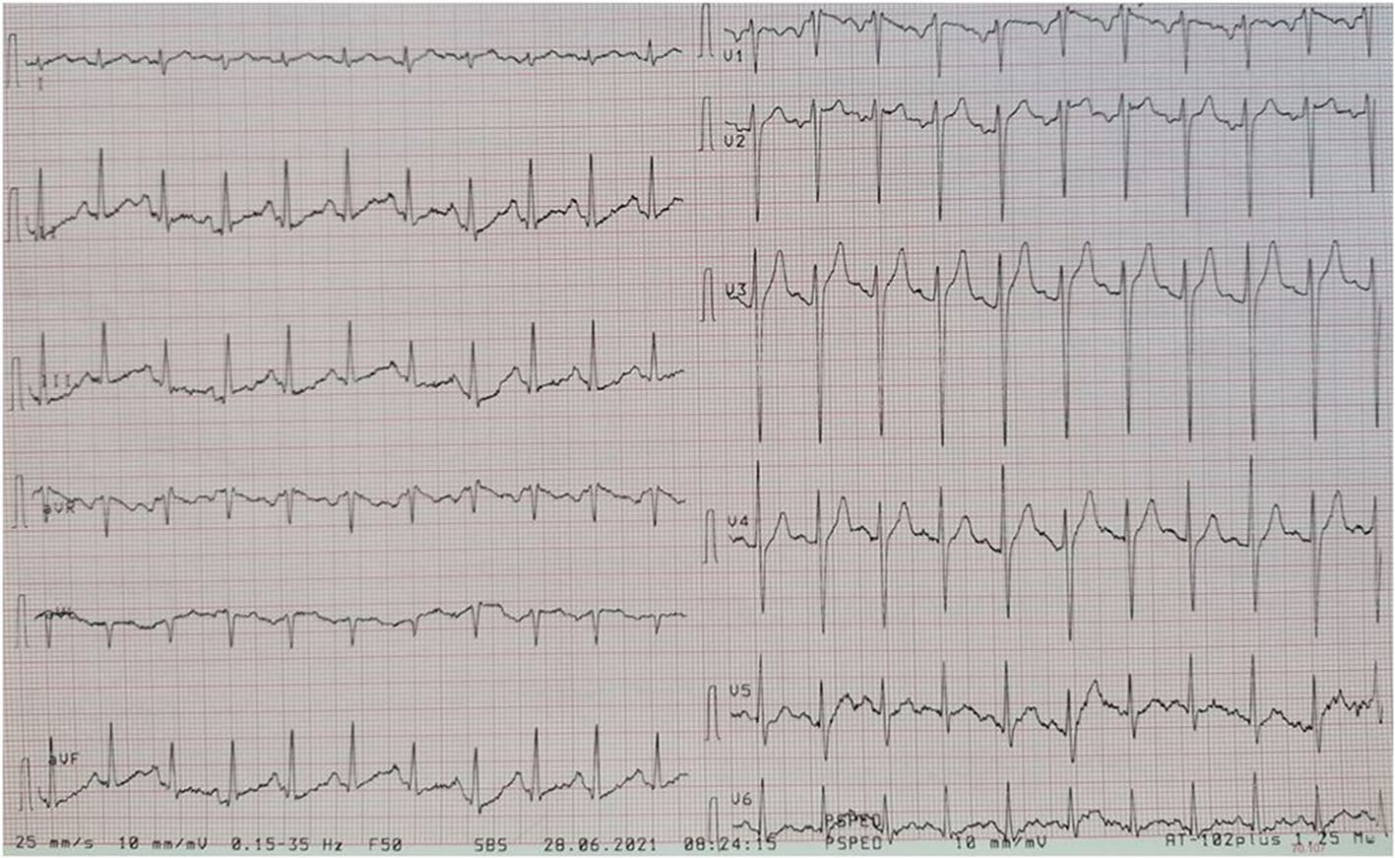
ECG

**Fig. 10:**
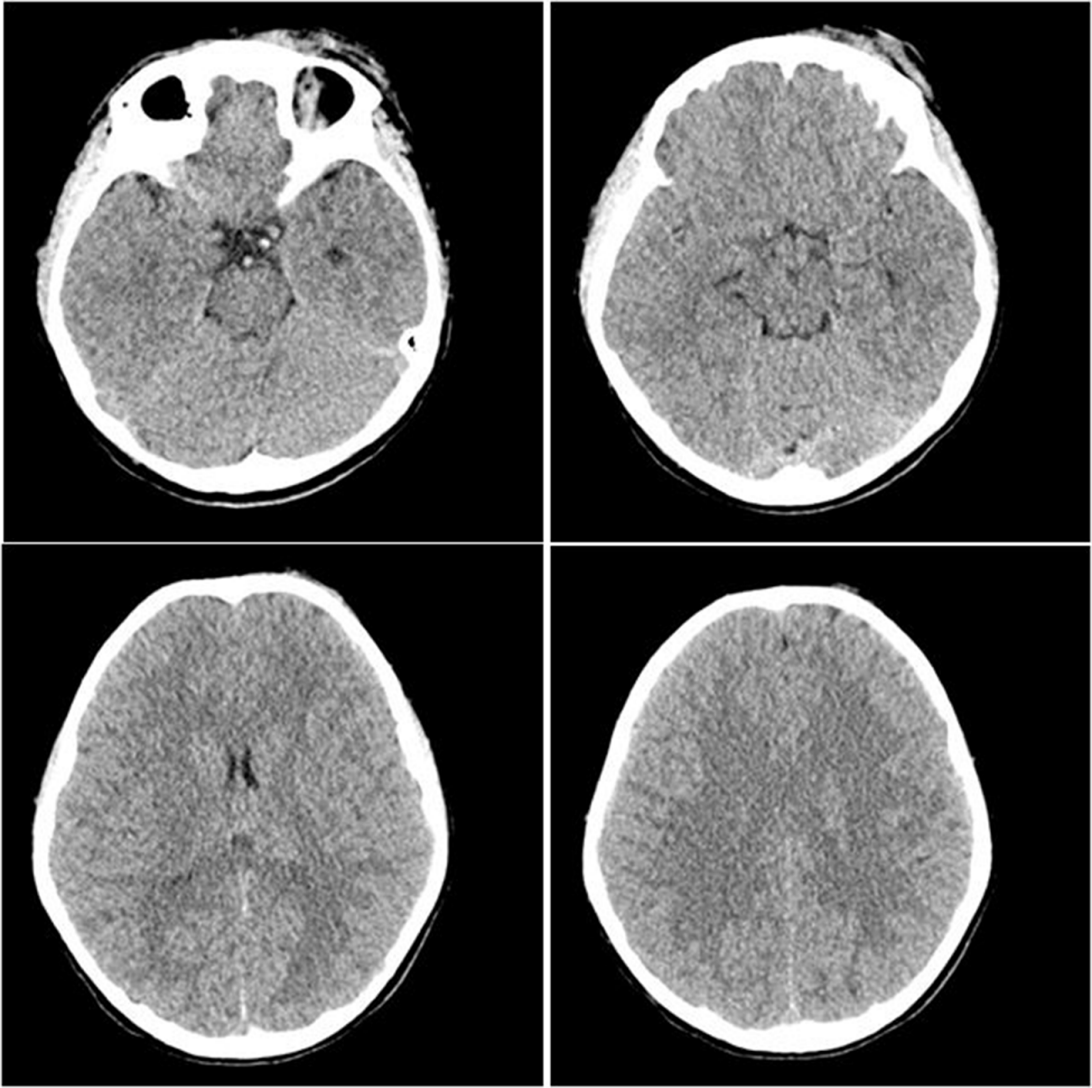
T0 brain CT

**Fig. 11:**
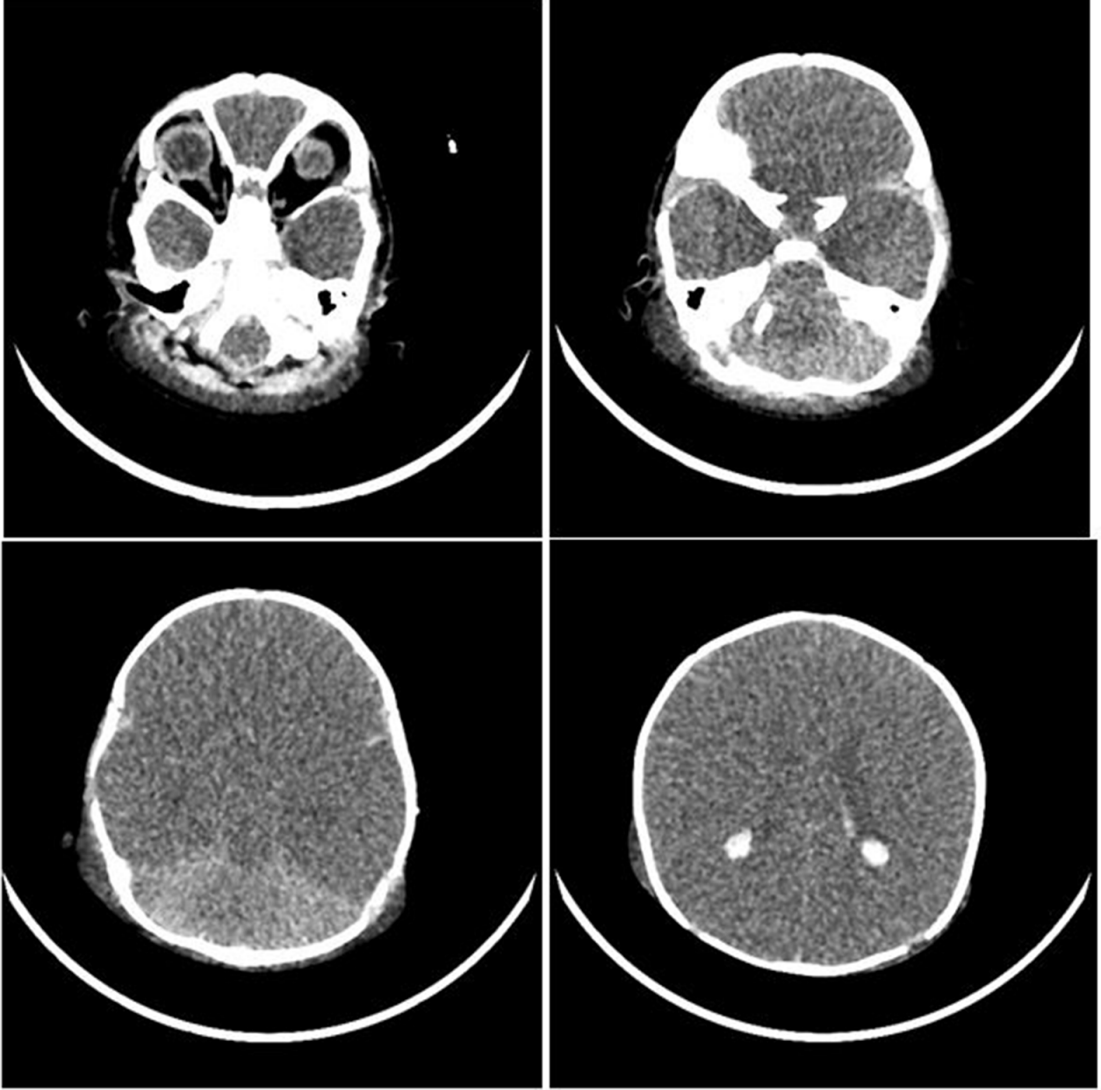
Cerebral edema brain CT

In the real world, the scenario would take 6 h, but in the simulation time transitions between one time point and the next one occurred once the team had performed the critical actions of that time or when they declared they would not perform any further action. Time transitions were announced by a microphone from the control room. If deviations from the protocol occurred, we provided minor scenarios evolving into complications: cerebral edema, hypoglycemia, hypokalemia (information regarding the various simulation times and deviation from the main scenario due to errors are described in Appendices E–I).

At the end of the simulation, a validated scenario evaluation questionnaire was provided [12] (Appendix J). The questionnaire was used to collect feedback from participants regarding the simulation. The questionnaire consisted of 13 statements that could be rated using a Likert scale (from 1: strongly disagree to 5: strongly agree), and three open-ended questions that allowed participants to give a freer evaluation of the scenario. In general, the questionnaire’s aims were to assess whether the scenario was realistic and useful for improving professional skills, the impact of the simulation on learning, the usefulness of debriefing, and how the scenario impacted certain skills.

### Statistical analysis

The scores for each item of the questionnaire are expressed as mean and standard deviation (SD). Groups were compared using the Mann–Whitney U test. A p-value < 0.05 was considered statistically significant. All analyses were performed using SPSS version 21.0 (IBM, New York, NY, USA).

### Briefing content

Mark is a 10-year-old boy, anamnestic weight 28 kg, who arrives at the emergency department accompanied by his mother. The mother reports that the child has had a cold in the previous few days and today has nasal congestion, vomiting, and is particularly tired. The mother also noticed that the child urinates a lot in the past few days. At first look, he appears tachypneic and dehydrated, with fuzziness. If requested by participants, other information can be provided as in Appendix C. Initial simulator settings and changes in vital parameters at different time points according to the actions performed by participants are reported in Appendix D.

## Results

The scenario was played by 78 pediatrics and 20 emergency medicine residents. At the end of the simulation, all participants filled the scenario evaluation questionnaire (Table 1).

**Table 1:** Scores (mean, SD) for the 13 items of the evaluation questionnaire, comparison between pediatrics and emergency medicine residents (by Mann-Withney U test)

Statement	All (*n* = 98)		Pediatrics residents (*n* = 78)		Emergency medicine residents (*n* = 20)		p-value
	Mean	St.Dev.	Mean	St.Dev.	Mean	St.Dev.	
1. This case presented during the simulation is relevant to my work	4.72	0.47	4.77	0.45	4.55	0.51	0.12
2. The simulation case was realistic	4.37	0.68	4.41	0.67	4.20	0.70	0.25
3. This simulation case was effective in teaching basic resuscitation skills	3.27	1.16	3.43	1.11	2.65	1.18	0.009*
4. The debrief promoted reflection and team discussion	4.49	0.74	4.53	0.76	4.33	0.62	0.19
5. The group discussion helped me develop and prioritize evaluation and management options for a child found to have new onset diabetes and DKA	4.41	0.67	4.46	0.64	4.19	0.75	0.20
6. The facilitators created a safe environment for discussion and exploration	4.34	0.82	4.56	0.53	3.35	1.11	< 0.0001*
7. Demonstrate ability to assess and emergently manage airway, breathing and circulation	2.88	0.92	3.00	0.87	2.40	0.99	0.03*
8. Formulate a list of possible diagnoses and prioritize elements of evaluation	3.24	0.76	3.36	0.74	2.80	0.70	0.007*
9. Identify laboratory abnormalities diagnostic of DKA	3.97	0.65	4.01	0.73	3.80	0.70	0.28
10. Manage fluid resuscitation and insulin administration in a pediatric patient with new onset diabetes in DKA	3.46	0.92	3.51	0.89	3.25	1.02	0.36
11. Identify risks, signs and symptoms of cerebral edema associated with DKA	3.12	0.97	3.19	0.90	3.85	1.18	0.18
12. Construct a disposition plan after stabilization in the emergency department for a pediatric patient in DKA	2.91	1.06	3.12	0.97	2.10	1.02	0.0004*
13. Utilize effective team leadership, roles and communication strategies	3.49	0.94	3.64	0.77	2.90	1.29	0.01*

*p < 0.05

Overall, the scenario was rated as realistic (mean score 4.37 ± 0.68, on a scale from 1 to 5) and relevant to professional training (mean score 4.72 ± 0.47), but less effective in teaching basic resuscitation techniques (mean score 3.27 ± 1.16). The debriefing, group discussion and facilitators’ activity were all rated positively (mean scores: 4.49 ± 0.74, 4.41 ± 0.67, and 4.34 ± 0.82, respectively).

The scenario appeared useful in increasing confidence in interpreting laboratory tests in cases of DKA (mean score 3.97 ± 0.65), group organization and communication strategies (mean score 3.49 ± 0.94), and managing the treatment of DKA (mean score 3.46 ± 0.92).

When comparing pediatrics residents and emergency medicine residents evaluations, pediatrics residents rated items number 3, 6, 7, 8, 12, and 13 significantly higher than emergency medicine residents.

**Fig. 12:**
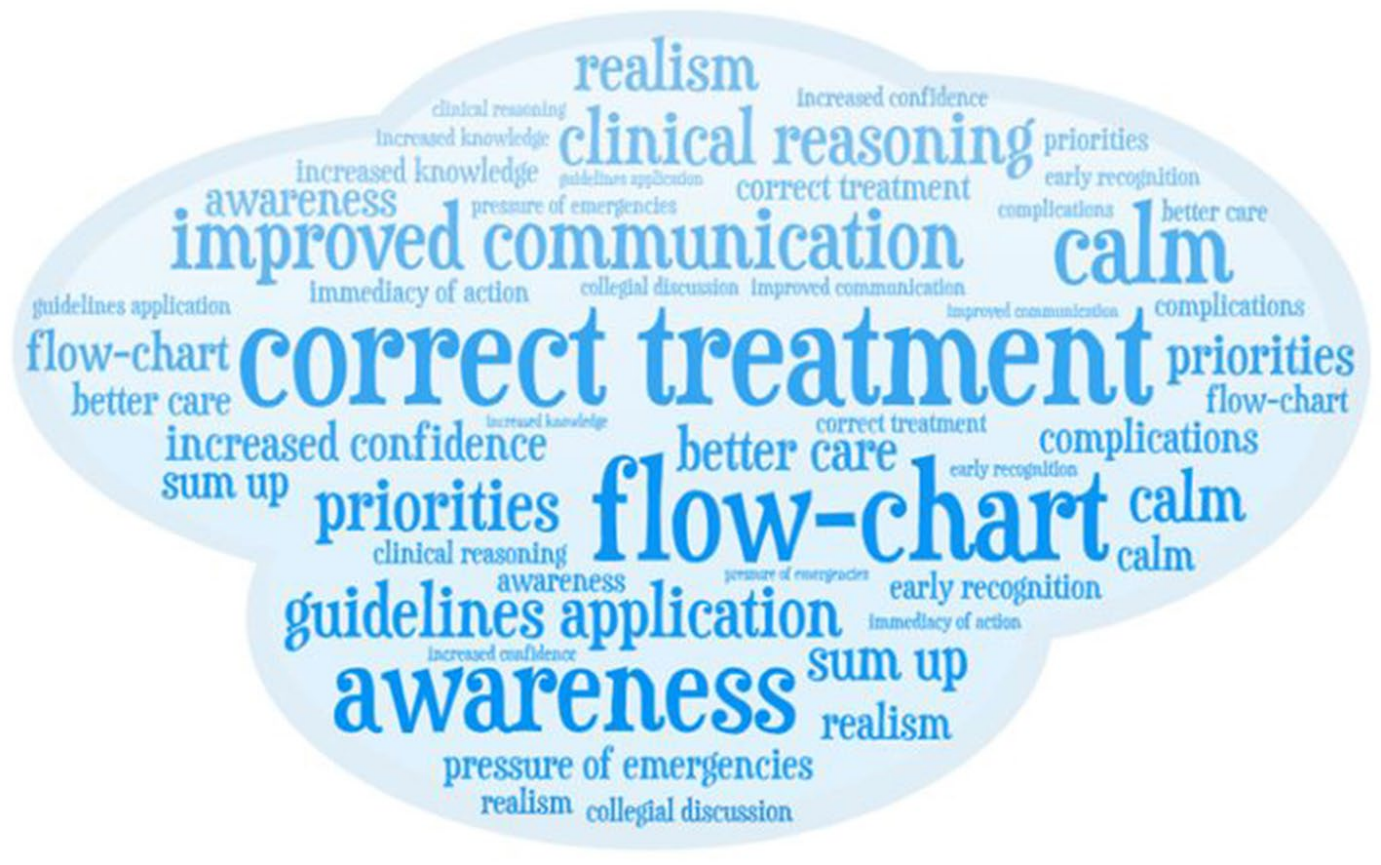
Cloud representation of the answers to the question: “Can you list/describe 1 or more ways this session will change how you do your job?”

When asked how this simulation would change the way they would work in the future, the most frequent responses were to pay more attention to guidelines, to have a flowchart to follow, and to pay more attention to the communication among colleagues and with nursing staff (in particular, the main problems were the lack of closed-loop communication, followed by generic and not individually addressed directives and instructions given by the team leader). (Fig. 12, in the word cloud the size of the word is proportional to their frequency).

When asked how the simulation could be improved, the main answers were: to set it up to give more time for clinical reasoning and to provide educational material on pediatric DKA in advance.

## Discussion

DKA is a frequent manifestation at the onset of T1DM in pediatric age, possibly associated with a wide range of complications, many of which may be iatrogenic in nature, as a consequence of wrong or delayed treatment. Thus, early diagnosis and proper treatment are strong predictors of positive outcomes, while deviations from guidelines are associated with a higher number of complications. As reported by Mendpara et al. [9] in the DKA management in a single center experience, at least one deviation from ISPAD 2018 guidelines was observed in 82.9% of cases and two or more errors were made in 40%, including: failure to administer the initial fluid bolus, expected in case of shock, in 5.7% of cases; excessive fluid administration in 11.4%; insufficient initial fluid administration in 35.7%; inadequate initial potassium supplementation in 18.6%; delayed response to falling serum potassium concentration (21.4%) and blood glucose (34.4%); erroneous insulin administration in 27.1%. The observed complications were hypokalemia (27.1%), hypoglycemia (35.7%), and cerebral edema (24.3%).

To possibly avoid such complications, teaching residents, who will be future clinicians directly involved in managing children with DKA, the proper management of pediatric DKA in a simulation setting seems to be a safe way to practice strict adherence to current international guidelines. In this regard, we develop a standardized scenario that aims to guide step-by-step the learners through the flowchart of DKA management and, at the same time, considers alternative evolutions in the case of possible deviations from guidelines.

We chose to test such a scenario not only with pediatrics residents, but also with emergency medicine residents, because not rarely the management of children with DKA occurs in emergency departments, and not only pediatricians but also emergency medicine physicians are involved in it. Due to its complex and risky management, direct exposure to real clinical situations alone is not sufficient to achieve adequate skills in pediatric DKA for residents, both in pediatrics and in emergency medicine. For these reasons, the use of simulation with the purpose of facilitating learning through immersion, reflection, feedback, and practice, without the risks of real-world experience, could prove useful.

Our study confirmed that this simulation scenario was on the whole positively judged in teaching the strict application of current guidelines on the management of pediatric DKA. It allowed participants to strengthen their confidence in interpreting laboratory data and setting rehydration and insulin therapy in the context of pediatric DKA. The simulation also allowed participants to develop non-technical skills (teamwork, leadership, communication, and clarity in assigning roles), and to understand the importance of accurate and effective communication among colleagues, with the nursing staff, and with parents in an emergency setting.

The main limitation of the described case scenario is its standardized structure, an intrinsic characteristic of simulation-based learning. Still, it can also be considered a point of strength, allowing the participants to become confident with the strict application of the algorithm, leaving variations to the case scenario (different severity and dehydration rates, patient weight and vitals…) to further steps of the teaching process.

## Conclusion

The use of a standardized scenario of pediatric DKA may be a valid tool to go through all the steps of pediatric DKA management and thus can be effectively used to reinforce theoretical knowledge in residents and to directly and safely practice pediatric DKA management.

### Electronic supplementary material

Below is the link to the electronic supplementary material.

Supplementary Material 1

Supplementary Material 2

Supplementary Material 3

Supplementary Material 4

Supplementary Material 5

Supplementary Material 6

Supplementary Material 7

Supplementary Material 8

Supplementary Material 9

Supplementary Material 10

Supplementary Material 11

Supplementary Material 12

## Data Availability

All data generated or analysed during this study are included in this published article [and its supplementary information files].

## Abbreviations

DKA: diabetic ketoacidosis
T1DM: type 1 diabetes mellitus
ISPAD: International Society for Pediatric and Adolescent Diabetes
