# Supplementary material for: Effectiveness of a standardized scenario in teaching the management of pediatric diabetic ketoacidosis (DKA) to residents: a simulation cross-sectional study
Source: BMC Med Educ. 2024 Mar 27;24:345. doi: 10.1186/s12909-024-05334-0 (PMC10976788; doi:10.1186/s12909-024-05334-0)
Supplement: Supplementary file 1 — Supplementary Material 1 [file 12909_2024_5334_MOESM1_ESM.docx]

| **APPENDIX A** | |
| --- | --- |
| **SIMULATION CASE TITLE: A CASE OF PEDIATRIC DKA**  **Learning objective and key actions** | |
| **Learning objectives and teaching characteristics of the scenario** | 1. Proper management in emergency of a case of pediatric DKA (fluids, potassium, insulin) 2. Possible recognition of signs and symptoms of cerebral edema and application of corrective actions   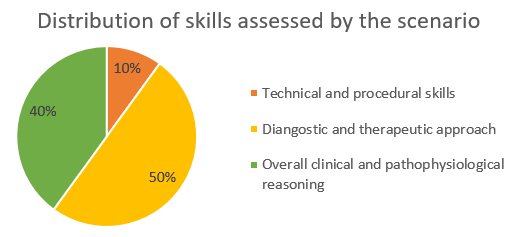 |
| **Key actions** | - Fluid management - Potassium management - Insulin management |
