# Supplementary material for: Effectiveness of a standardized scenario in teaching the management of pediatric diabetic ketoacidosis (DKA) to residents: a simulation cross-sectional study
Source: BMC Med Educ. 2024 Mar 27;24:345. doi: 10.1186/s12909-024-05334-0 (PMC10976788; doi:10.1186/s12909-024-05334-0)
Supplement: Supplementary file 2 — Supplementary Material 2 [file 12909_2024_5334_MOESM2_ESM.docx]

| **APPENDIX B** | |
| --- | --- |
| **SIMULATION CASE TITLE: A CASE OF PEDIATRIC DKA**  **Equipment / Environment** | |
| **Materials needed** | - Emergency simulation room - High-fidelity pediatric simulator - PALS cart - Monitor - Infusion pumps - Glucometer with ketone measurement - Ketone strips (ketonuria and ketonemia) - Blood glucose strips - Urine sticks - Broselow tape - Flashlight - PPE (staff gowns, gloves, mask and face shield) - Blood chemistry tests - BGA - ECG - Chest X-ray - CT brain scan |
| **Medications** | - Pediatric resuscitation medication - Saline, glucose solution, ringer lactate - Potassium chloride and phosphate - Insulin - Sodium bicarbonate - Mannitol - Ondansetron - Others |
| **Embedded participants** | - Mother or father - 2 nurses |
