# Supplementary material for: Effectiveness of a standardized scenario in teaching the management of pediatric diabetic ketoacidosis (DKA) to residents: a simulation cross-sectional study
Source: BMC Med Educ. 2024 Mar 27;24:345. doi: 10.1186/s12909-024-05334-0 (PMC10976788; doi:10.1186/s12909-024-05334-0)
Supplement: Supplementary file 3 — Supplementary Material 3 [file 12909_2024_5334_MOESM3_ESM.docx]

| **APPENDIX C** | |
| --- | --- |
| **SIMULATION CASE TITLE: A CASE OF PEDIATRIC DKA**  **The initial scenario** | |
| **Description of scenario** | Mark is a 10-year-old boy who has been presenting with nasal congestion for a few days; nonblood and non-biliary vomiting, and asthenia for one day. Reduced feeding, active diuresis, no fever, no diarrhea.  On arrival in the emergency department, he presents tachypnoic and dehydrated.  Recent history:   - Rhinitis - Polyuria in previous days - Weight loss of 0.5-1 kg (5-10% of body weight), last weight from his family doctor 28 kg   Past medical history: nothing relevant |
| **SAMPLE** | - Signs and symptoms: increased diuresis in the last three days, 0.5-1 kg weight loss in the last week - Allergies: none - Medications: acetaminophen as needed - Concomitant or past medical conditions: none - Last meal: milk one hour before arrival in ER - Triggering event: as above |
| **ROS (revision of systems)** | Negative except for that already reported. |
| **Family history** | Not significant. |
| **Social history** | He lives with his parents and grandmother. He is cared for by his grandmother during the day. |
