# Supplementary material for: Effectiveness of a standardized scenario in teaching the management of pediatric diabetic ketoacidosis (DKA) to residents: a simulation cross-sectional study
Source: BMC Med Educ. 2024 Mar 27;24:345. doi: 10.1186/s12909-024-05334-0 (PMC10976788; doi:10.1186/s12909-024-05334-0)
Supplement: Supplementary file 4 — Supplementary Material 4 [file 12909_2024_5334_MOESM4_ESM.docx]

| **APPENDIX D** | |
| --- | --- |
| **SIMULATION CASE TITLE: A CASE OF PEDIATRIC DKA**  **How the patient presents at T0** | |
| **Vital parameters** | - BP: 100/53 mmHg - HR: 130 bpm - RR: 43 acts/minute - T: 36.8°C - Weight: 28 kg reported |
| **Venous accesses** | To be placed. |
| **Monitor** | To be placed. |
| **Physical examination** | - General: poor general condition, tachypneic, moderate respiratory distress, rhinorrhea - Pupils: iso/iso - Neck: no meningeal signs - Chest: VM normotransmitted, tachypneic with diaphragmatic reentry - Cardiovascular: valid tones, rhythmic, tachycardic, no murmurs, peripheral pulses normal, no edema, - Refill: 3-4" - Abdomen: treatable, poorly evaluable due to poor compliance - Skin: normothermic, dry, no rush - Extremities: cold - Neurological:   - AVPU: V   - GCS: 11   - Soporous, awakenable upon verbal stimulus. |
