# Supplementary material for: Effectiveness of a standardized scenario in teaching the management of pediatric diabetic ketoacidosis (DKA) to residents: a simulation cross-sectional study
Source: BMC Med Educ. 2024 Mar 27;24:345. doi: 10.1186/s12909-024-05334-0 (PMC10976788; doi:10.1186/s12909-024-05334-0)
Supplement: Supplementary file 5 — Supplementary Material 5 [file 12909_2024_5334_MOESM5_ESM.docx]

| **APPENDIX F** | | | |
| --- | --- | --- | --- |
| **SIMULATION CASE TITLE: A CASE OF PEDIATRIC DKA**  **Scenario A (main scenario) progression** | | | |
| **TIME** | **ACTIONS TO BE PERFORMED** | **PATIENT CONDITION AND EXAMINATIONS**  **(To be provided only if requested)** | **ERRORS** |
| **T0** | - Collect the patient’s history - First assessment - Monitor - Place two peripheral venous accesses - Blood tests - BGA - Blood glucose - Ketonemia | Glucose: 25 mmol/L  ≈ 451 mg/dL  KET: 5.5 mmol/L  EGA:  - pH: 7.11  - pCO2: 15 mmHg  - pO2: 80 mmHg  - HCO_3_^-^: 4.9 mmol/L  - BE: -22.1 mmol/L  - Sodium: 132 mEq/L  - Corrected Na^+^: 138 mEq/L  - Potassium: 3.5 mEq/L  - Corrected K^+^: 1.76 mEq/L  - Bicarbonate: 5 mmol/L  - Ionized ca^2+^: 1.44 mg/dL  - Magnesium: 1.5 mEq/L  - Phosphorus: 3.2 mEq/L  - Lactate: 1.8 mEq/L  WBC: 18 x 10^9^/L  Hb: 157 g/L  15.7 g/dL  Htc: 0.47  PLT: 216 x 10^9^/L  BUN: 7.1 mmol/L  20 mg/dL  Creatinine: 53 µmol/L  0.6 mg/dL  Urinalysis: ketones 3+  glucose 3+ | - Don't ask for: glycemia, ketonemia, BGA |
|  | - **DD, identify DKA** - **START TREATMENT**   **Saline solution 0.9% 280 mL/h (10-20 mL/Kg/h)**  **+ K^+^ 20 mEq/L*** |  | - Wrong fluid - Wrong hydration rate (excess or poor fluid) - Failure to start potassium infusion - Bolus bicarbonate - Bolus insulin SC/IV |
| **T1A**  **(60 m)** | Monitoring:  - Vital parameters and GCS  - Fluid input and output  - Blood glucose  - Ketonemia | HR: 130 bpm  RR: 31 acts/minute  DTX: 25 mmol/L  ≈ 451 mg/dL  KET: 5.5 mmol/L |  |
| **T2A**  **(2 h)** | Monitoring:  - Vital parameters and GCS  - Fluid input and output  - Blood glucose  - BGA | HR: 130 bpm  RR: 31 acts/minute  Glucose: 21.7 mmol/L  390 mg/dL  BGA:  - pH: 7.00  - pCO2: 15mmHg  - HCO3-: 6.5mmol/L |  |
|  | **Infusion modification:**  **Saline solution 0.9% at 100 mL/h**  **+ K^+^ 40 mEq/L***  **Human-regular INSULIN**  **0.05-0.1 U/Kg/h** |  | - Insulin startup timing - Wrong type of insulin - Bolus insulin |
| **T3A**  **(3 h)** | Monitoring:  - Vital parameters and GCS  - Fluid input and output  - Blood glucose  - Ketonemia | HR: 127 bpm  RR: 29 acts/minute  Glucose: 19.44 mmol/L  350 mg/dL  KET: 4.5 mmol/L |  |
| **T4A**  **(4h)** | Monitoring:  - Vital parameters and GCS  - Fluid input and output  - Blood glucose  - BGA | HR: 110 bpm  RR: 20 acts/minute  Glucose: 15.56 mmol/L  280 mg/dL  BGA:  - pH: 7.15  - pCO2: 20 mmHg  - HCO3-: 8 mmol/L  - K+: 3 mEq/L |  |
|  | **Infusion modification:**  **With two-bag system:**   - **Saline solution 0.9% at 50 mL/h + K^+^ 40 mEq/L*** - **Glucose solution 10% at 50 mL/h + K^+^ 40 mEq/L***   **With one-bag system:**   - **Glucose solution 5% at 100 mL/h + NaCl 0.45% + K^+^ 40 mEq/L ***   **-Human-regular INSULIN 0.05-0.1 U/Kg/h** |  | - Lack of glucose startup |
| **T5A**  **(5 h)** | Monitoring:  - Vital parameters and GCS  - Fluid input and output  - Blood glucose  - Ketonemia | HR: 110 bpm  RR: 20 acts/minute  Glucose: 8.89 mmol/L  160mg/dL  KET: 3 mmol/L |  |
| **T6A**  **(6 h)** | Monitoring:  - Vital parameters and GCS  - Fluid input and output  - Blood glucose  - BGA | HR: 102 bpm  RR: 20 acts/minute  Glucose: 8.17 mmol/L  147 mg/dL  EGA:  - pH: 7.2  - pCO2: 25 mmHg  - HCO3-: 12 mmol/L |  |
|  | **Infusion modification:**  **With two-bag system:**   - **Saline solution 0.9% at 50 mL/h + K^+^ 40 mEq/L*** - **Glucose solution 20% at 50 mL/h + K^+^ 40 mEq/L***   **With one-bag system:**   - **Glucose solution 10% at 100 mL/h + NaCl 0.45% + K^+^ 40 mEq/L***   **- INSULIN 0.05-0.1 U/Kg/h** |  |  Failure to increase % of glucose solution   Insulin reduction |
| *50% KCl and 50% K phosphate | | | |
