# Supplementary material for: Effectiveness of a standardized scenario in teaching the management of pediatric diabetic ketoacidosis (DKA) to residents: a simulation cross-sectional study
Source: BMC Med Educ. 2024 Mar 27;24:345. doi: 10.1186/s12909-024-05334-0 (PMC10976788; doi:10.1186/s12909-024-05334-0)
Supplement: Supplementary file 6 — Supplementary Material 6 [file 12909_2024_5334_MOESM6_ESM.docx]

| **APPENDIX G** | | | |
| --- | --- | --- | --- |
| **SIMULATION CASE TITLE: A CASE OF PEDIATRIC DKA**  **Scenario B (cerebral edema) progression** | | | |
| **Mistakes that lead to the initiation of this scenario** | | - Non-recognition of DKA - Excess fluid (>20 mL/Kg/h or >4 L/m2/24h) or hypotonic solution - Insulin bolus SC/IV (glucose becomes: 11.39 mmol/L   205 mg/dL)   - HCO3- bolus (serum bicarbonate becomes 15 mmol/L) - Drop in blood glucose > 5.56 mmol/L/h (100 mg/dL/h) | |
|  | | | |
| **TIME** | **ACTIONS TO BE PERFORMED** | **PATIENT CONDITION** | **ERRORS** |
| **T1B** | Recognition of cerebral edema  Implementation of corrective measures:   - Head lifting - Hypertonic solution or mannitol - Reducing fluid infusion rate - Brain imaging (if not delaying treatment) - Neurosurgeon consultation | Onset of cerebral edema:   - 80bpm bradycardia - Impaired neurological or convulsions: irritable, worsening vomiting, worsening GCS: 9 - BP 125/90 mmHg - RR:31 acts/minute | - Non-recognition of cerebral edema |
