# Supplementary material for: Effectiveness of a standardized scenario in teaching the management of pediatric diabetic ketoacidosis (DKA) to residents: a simulation cross-sectional study
Source: BMC Med Educ. 2024 Mar 27;24:345. doi: 10.1186/s12909-024-05334-0 (PMC10976788; doi:10.1186/s12909-024-05334-0)
Supplement: Supplementary file 7 — Supplementary Material 7 [file 12909_2024_5334_MOESM7_ESM.docx]

| **APPENDIX H** | | | |
| --- | --- | --- | --- |
| **SIMULATION CASE TITLE: A CASE OF PEDIATRIC DKA**  **Scenario C (hypoglycemia) progression** | | | |
| **Mistakes that lead to the initiation of this scenario** | | - DKA recognition  - Failure to insert glucose → glucose: 6.67 mmol/L  120mg/dL | |
|  | | | |
| **TIME** | **ACTIONS TO BE PERFORMED** | **PATIENT CONDITION AND EXAMINATIONS** | **ERRORS** |
| **T4C** | Insert glucose solution | Hypoglycemia | - Not inserting glucose solution |
| **T5C** | Non-recognition  END OF SIMULATION |  |  |
