# Supplementary material for: Effectiveness of a standardized scenario in teaching the management of pediatric diabetic ketoacidosis (DKA) to residents: a simulation cross-sectional study
Source: BMC Med Educ. 2024 Mar 27;24:345. doi: 10.1186/s12909-024-05334-0 (PMC10976788; doi:10.1186/s12909-024-05334-0)
Supplement: Supplementary file 8 — Supplementary Material 8 [file 12909_2024_5334_MOESM8_ESM.docx]

| **APPENDIX I** | | |
| --- | --- | --- |
| **SIMULATION CASE TITLE: A CASE OF PEDIATRIC DKA**  **Scenario C (hypokalemia) progression** | | |
| **Mistakes that lead to the initiation of this scenario** | | - Failure to correct K+ |
|  | | |
| **TIME** | **ACTIONS TO BE PERFORMED** | **PATIENT CONDITION AND EXAMINATIONS** |
| **T3D** | Recognize arrhythmic pattern → start K infusion → start CPR if cardiac arrest | After 4 hours → arrhythmia (ventricular tachycardia with pulse) → cardiac arrest (pulseless ventricular tachycardia) → END OF SIMULATION |
