# Supplementary material for: Effectiveness of a standardized scenario in teaching the management of pediatric diabetic ketoacidosis (DKA) to residents: a simulation cross-sectional study
Source: BMC Med Educ. 2024 Mar 27;24:345. doi: 10.1186/s12909-024-05334-0 (PMC10976788; doi:10.1186/s12909-024-05334-0)

APPENDIX E

SIMULATION CASE TITLE: A CASE OF PEDIATRIC DKA

Scenario summary diagram and transition between simulation times

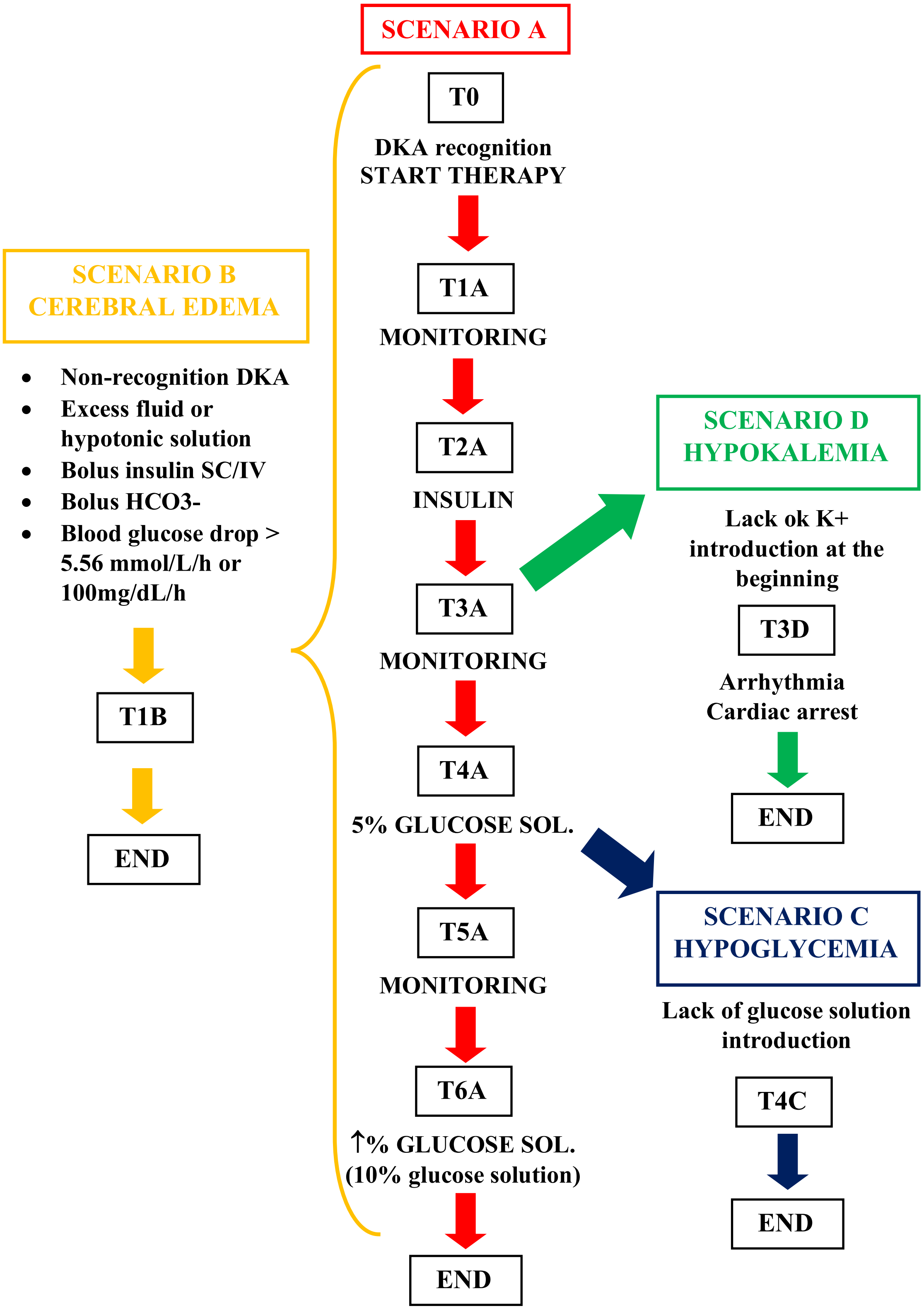

Supplement: Supplementary file 10 — Supplementary Material 10 [file 12909_2024_5334_MOESM10_ESM.pdf]
