# Supplementary material for: Effectiveness of a standardized scenario in teaching the management of pediatric diabetic ketoacidosis (DKA) to residents: a simulation cross-sectional study
Source: BMC Med Educ. 2024 Mar 27;24:345. doi: 10.1186/s12909-024-05334-0 (PMC10976788; doi:10.1186/s12909-024-05334-0)
Supplement: Supplementary file 11 — Supplementary Material 11 [file 12909_2024_5334_MOESM11_ESM.docx]

| **APPENDIX J** | | | | | | | | | | |
| --- | --- | --- | --- | --- | --- | --- | --- | --- | --- | --- |
| **SIMULATION CASE TITLE: A CASE OF PEDIATRIC DKA**  **Ped DKA evaluation form**  (Roberts C, Keilman A, Pearce J, et al. Pediatric Emergency Medicine Didactics and Simulation (PEMDAS): Pediatric Diabetic Ketoacidosis. MedEdPORTAL. 2021;17:11098) | | | | | | | | | | |
|  | Strongly  Disagree | | Disagree | | Neutral | | Agree | | Strongly  Agree | |
| 1. This case presented during the simulation is relevant to my work. | 1 | 2 | | 3 | | 4 | | 5 | |  |
| 1. The simulation case was realistic. | 1 | 2 | | 3 | | 4 | | 5 | |  |
| 1. This simulation case was effective in teaching basic resuscitation skills. | 1 | 2 | | 3 | | 4 | | 5 | |  |
| 1. The debrief promoted reflection and team discussion. | 1 | 2 | | 3 | | 4 | | 5 | |  |
| 1. The group discussion helped me develop and prioritize evaluation and management options for a child found to have new onset diabetes and DKA. | 1 | 2 | | 3 | | 4 | | 5 | |  |
| 1. The facilitators created a safe environment for discussion and exploration. | 1 | 2 | | 3 | | 4 | | 5 | |  |

After participating in this session, how confident are you in your ability to:

|  | Very Unconfident | Unconfident | Neutral | Confident | Very Confident |
| --- | --- | --- | --- | --- | --- |
| Demonstrate ability to assess and emergently manage airway, breathing and circulation | 1 | 2 | 3 | 4 | 5 |
| Formulate a list of possible diagnoses and prioritize elements of evaluation | 1 | 2 | 3 | 4 | 5 |
| Identify laboratory abnormalities diagnostic of DKA | 1 | 2 | 3 | 4 | 5 |
| Manage fluid resuscitation and insulin administration in a pediatric patient with new onset diabetes in DKA | 1 | 2 | 3 | 4 | 5 |
| Identify risks, signs and symptoms of cerebral edema associated with DKA | 1 | 2 | 3 | 4 | 5 |
| Construct a disposition plan after stabilization in the emergency department for a pediatric patient in DKA | 1 | 2 | 3 | 4 | 5 |
| Utilize effective team leadership, roles and communication strategies | 1 | 2 | 3 | 4 | 5 |

Can you list/describe 1 or more ways this session will change how you do your job?

How can we improve this simulation?

Additional Comments:
