# Supplementary material for: Effectiveness of a standardized scenario in teaching the management of pediatric diabetic ketoacidosis (DKA) to residents: a simulation cross-sectional study
Source: BMC Med Educ. 2024 Mar 27;24:345. doi: 10.1186/s12909-024-05334-0 (PMC10976788; doi:10.1186/s12909-024-05334-0)
Supplement: Supplementary file 12 — Supplementary Material 12 [file 12909_2024_5334_MOESM12_ESM.docx]

**List of additional files:**

**Additional file 1** (.doc): Appendix A

**Additional file 2** (.mp4):Video recording of the setting of the scenario

**Additional file 3** (.doc): Appendix B

**Additional file 4** (.doc): Appendix C

**Additional file 5** (.doc): Appendix D

**Additional file 6** (.doc): Appendix E

**Additional file 7** (.doc): Appendix F

**Additional file 8** (.doc): Appendix G

**Additional file 9** (.doc): Appendix H

**Additional file 10** (.doc): Appendix I

**Additional file 11** (.doc): Appendix J
